# Risk Factors for Cervical Precancer and Cancer in HIV-Infected, HPV-Positive Rwandan Women

**DOI:** 10.1371/journal.pone.0013525

**Published:** 2010-10-20

**Authors:** Kathryn Anastos, Donald R. Hoover, Robert D. Burk, Antonio Cajigas, Qiuhu Shi, Diljeet K. Singh, Mardge H. Cohen, Eugene Mutimura, Charles Sturgis, William C. Banzhaf, Philip E. Castle

**Affiliations:** 1 Departments of Medicine and Epidemiology and Population Health, Montefiore Medical Center and Albert Einstein College of Medicine, Bronx, New York, United States of America; 2 Department of Statistics and Institute for Health, Health Care Policy and Aging Research, Rutgers University, Piscataway, New Jersey, United States of America; 3 Division of Genetics, Department of Pediatrics, Albert Einstein College of Medicine, Bronx, New York, United States of America; 4 Department of Pathology, Montefiore Medical Center and Albert Einstein College of Medicine, Bronx, New York, United States of America; 5 Department of Epidemiology and Community Health, New York Medical College, Valhalla, New York, United States of America; 6 Feinberg School of Medicine, Northwestern University, Chicago, Illinois, United States of America; 7 John Stroger (formerly Cook County) Hospital and Rush University, Chicago, Illinois, United States of America; 8 Women's Equity in Access to Care and Treatment, Kigali, Rwanda; 9 Providence Regional Medical Center, Everette, Washington, United States of America; 10 University of Chicago Pritzker School of Medicine, Evanston, Illinois, United States of America; 11 National Cancer Institute, National Institutes of Health, Department of Health and Human Services, Bethesda, Maryland, United States of America; Instituto de Pesquisa Clinica Evandro Chagas, FIOCRUZ, Brazil

## Abstract

**Background:**

Although cervical cancer is an AIDS-defining condition, infection with human immunodeficiency virus (HIV) may only modestly increase the risk of cervical cancer. There is a paucity of information regarding factors that influence the natural history of human papillomavirus (HPV) in HIV-infected women. We examined factors associated with cervical intraepithelial neoplasia grade 3 or cancer (CIN3+) in Rwandan women infected with both HIV and HPV (HIV+/HPV+).

**Methods:**

In 2005, 710 HIV+ Rwandan women ≥25 years enrolled in an observational cohort study; 476 (67%) tested HPV+. Each woman provided sociodemographic data, CD4 count, a cervical cytology specimen and cervicovaginal lavage (CVL), which was tested for >40 HPV genotypes by MY09/MY11 PCR assay. Logistic regression models calculated odds ratios (OR) and 95% confidence intervals (CI) of associations of potential risk factors for CIN3+ among HIV+/HPV+ women.

**Results:**

Of the 476 HIV+/HPV+ women 42 (8.8%) were diagnosed with CIN3+. Factors associated with CIN3+ included ≥7 (vs. 0-2) pregnancies, malarial infection in the previous six months (vs. never), and ≥7 (vs. 0-2) lifetime sexual partners. Compared to women infected by non-HPV16 carcinogenic HPV genotypes, HPV16 infection was positively associated and non-carcinogenic HPV infection was inversely associated with CIN3+. CD4 count was significantly associated with CIN3+ only in analyses of women with non-HPV16 carcinogenic HPV (OR = 0.62 per 100 cells/mm^3^, CI = 0.40-0.97).

**Conclusions:**

In this HIV+/HPV+ population, lower CD4 was significantly associated with CIN3+ only in women infected with carcinogenic non-HPV16. We found a trend for higher risk of CIN3+ in HIV+ women reporting recent malarial infection; this association should be investigated in a larger group of HIV+/HPV+ women.

## Introduction

Cervical infections by carcinogenic human papillomavirus (HPV) genotypes cause virtually all cervical cancer and its immediate precursor lesion, cervical intraepithelial neoplasia grade 3 (CIN3) [1]–[3]. Exposure to genital HPV is nearly universal in sexually active people but the majority of infections clear within 1–2 years [4]–[6]. Persistence in women of a carcinogenic HPV type (HR-HPV) increases the risk of cervical intraepithelial neoplasia grade 3 (CIN3) and cancer [7]. A significant proportion of CIN3 will invade if not detected and treated [8].

Evidence suggests that secondary factors, or HPV cofactors, influence the progression of HR-HPV infections to CIN3 and cancer (CIN3+), including exogenous factors such as parity, smoking tobacco and possibly oral contraceptive use [9]. Co-infection by human immunodeficiency virus (HIV) may moderately increase the risk of cervical cancer [10], as immunosuppression may compromise the ability of the host to clear the HPV infection. The lack of a more profound impact of HIV on cervical cancer rates may be explained by two findings: 1) the early natural history of HPV16, the HPV genotype most often associated with progression to carcinoma, appears to be minimally affected by the severity of immunosuppression, suggesting it may more effectively escape immune surveillance even in an immunocompetent host [11]–[13]; and 2) increasing severity of immunosuppression only modestly increases the likelihood of HPV persistence [12]. However, there is a paucity of information regarding factors that influence the natural history of HPV in HIV-infected women.

We conducted a cross-sectional analysis of risk factors associated with CIN3 and cancer in a population of HIV-infected Rwandan women. We previously found in this population that HPV prevalence and presence of cytologic abnormalities were inversely associated with CD4 cell counts [13]. We limited the study population to HIV-infected women with prevalent HPV, since HR-HPV infection is required for the development of CIN3. CIN3 was our primary endpoint because CIN3 is the best surrogate for cancer risk and while CIN2 is the clinical threshold for treatment, it is a poorly reproducible diagnosis of cervical precancer [14]–[17].

## Methods

### Ethics Statement

Each participant provided written informed consent after viewing a video demonstrating study procedures. The Rwanda National Ethics Committee and the Institutional Review Board of Montefiore Medical Center, Bronx, NY approved the study protocol and the consent process.

### Participants

The Rwanda Women's Interassociation Study and Assessment (RWISA) is an observational cohort study of 710 HIV-infected and 226 HIV-uninfected Rwandan women enrolled May-November, 2005. Methods have been previously described in detail [13]. This analysis includes the 476 women co-infected with HIV and HPV (HIV+/HPV+). At study entry, participants provided historical information on sociodemographic characteristics, physical examination was performed, and blood specimens were taken for CD4 cell count, full blood count, and other laboratory testing.

Participants underwent pelvic examinations. To minimize contamination of gynecologic specimens by blood, exfoliated cervical cells (used for HPV DNA testing) were obtained by cervicovaginal lavage (CVL) prior to collection of a cervical cytology specimen, as previously described [13]. Colposcopy was provided to all women with any abnormal cytologic finding including atypical cells of uncertain significance and cervical biopsies were obtained for specific clinical indications: any visible lesion, suspected malignancy, or cytologic interpretation of high-grade squamous intraepithelial lesions (HSIL), atypical squamous cells of uncertain significance with suspicion of HSIL, atypical glandular cells, or cancer.

### Clinical Laboratory Data

CD4 counts were measured at the National Reference Laboratory of Rwanda by FACS Count (Becton Dickinson, San José, CA).

### HPV Testing

HPV testing was performed on CVL specimens taken at the enrollment visit.

In brief, 100 µl of each CVL specimen was mixed (1∶1) with a 2× solution of K buffer (containing Proteinase K at 400 g/mL, 2 mM EDTA, 2% Laureth-12, 100 mM Tris, pH 8.5) and incubated at 55°C for 2 hours followed by a further incubation at 95°C for 10 minutes. After the CVL specimens were digested with Proteinase K, 10 µL of each cell digest was taken to detect HPV DNA using the L1 MY09/MY11 modified PCR system with AmpliTaq Gold polymerase as described elsewhere [18]. Amplification products were probed for the presence of any HPV DNA by Southern blot with a radiolabeled generic probe mixture and subsequently typed by dot blot hybridization for HPV 6, 11, 13, 16, 18, 26, 31–35, 39, 40, 42, 45, 51–59, 61, 62, 64, 66–74, 81–85, 89, and 97. HPV16, 18, 31, 33, 35, 39, 45, 51, 52, 56, 58, 59, 66 and 68 were considered the primary carcinogenic HPV genotypes.

### Histopathology

Biopsies and excised tissue were read independently and blindly by pathologists at Montefiore Medical Center in the Bronx, New York or Evanston Hospital in Chicago Illinois, and were categorized as Negative, CIN1, CIN2, CIN3, and cancer. We used the most severe histopathologic diagnosis as the final participant diagnosis. Among the 202 biopsies, 3 were diagnosed as cancer, 39 CIN3, 21 CIN2, and 76 CIN1.

### Statistical Analysis

Of the 710 HIV-infected women 476 (67.0%) tested positive for any HPV DNA. Although non-carcinogenic HPV has not been shown to be a cause of cervical cancer, we included those women positive for non-carcinogenic HPV because some HPV genotypes categorized as non-carcinogenic HPV may be borderline carcinogenic [19] and may cause CIN3 (with little chance of invasion) particularly in HIV+ women [20]. We performed analyses on all HPV+ women, women with carcinogenic HPV genotypes and women with carcinogenic HPV genotypes *other than* HPV16.

Standard contingency table methods, with exact tests and when appropriate Mantel extension trend tests assessed univariate associations of categorical variables with CIN3 and cancer versus <CIN3. Two-sided P values <0.05 were considered statistically significant. The associations of ordinally categorized continuous variables with grade of neoplasia were assessed with logistic regression and trend tests. Odds ratios (OR) and 95% confidence intervals (CI) adjusted for variables identified in univariate analyses were calculated using logistic regression. Multivariate logistic regression models were fit using variables that had statistically significant (P≤0.05) unadjusted associations with CIN3+ and women that had complete information on these variables: 454 of 476 (95.4%) women positive for any HPV; 303 of 316 (95.9%) positive for carcinogenic HPV; and 241 of 251 (96.0%) women with non-16 carcinogenic HPV. Because of the relationship of CD4 counts with immunosuppression, all models included CD4 counts. We repeated these analyses with the outcome defined as CIN2 or more severe (CIN2+) (n = 63), including 21 women with CIN2, an equivocal precancer diagnosis.

## Results

Demographic and clinical characteristics of the included women are shown in **Table 1** **.** Of the 476 HPV+ women, 223 had a colposcopy and 202 had a cervical biopsy. Among women diagnosed with CIN3+ (n = 42; 39 CIN3, 3 cancer) the most common HPV types detected were HVP16 (33%), HPV58 (26%), HPV31 and 33 (21% each) and HPV 35 and 51 (19% each). HPV52 was harbored by 4.8% and 64.3% were infected with multiple HPV types, compared to 11.1% and 49.8% of women with <CIN3, respectively. Compared to women with a less severe histologic diagnosis, women diagnosed with CIN3+ reported more pregnancies (p_trend_ <0.001) and lifetime sexual partners (p_trend_  = 0.03), shared their residence with more people (p_trend_  = 0.01), had a higher income (p_trend_  = 0.02), were more likely to have had a recent malarial infection (p_trend_ = 0.047) and to harbor carcinogenic HPV (p_trend_ <0.001). CD4 counts were not related to cervical histologic findings among all HIV+/HPV+ women.

**Table 1:** Demographic and clinical characteristics in human immunodeficiency virus-infected, human papillomavirus (HPV)-infected women with and without cervical intraepithelial neoplasia grade 3 (CIN3) or cancer (CIN3+).

		All (N = 476)		CIN3+ (n = 42)		<CIN3 (n = 434)		CIN3+ vs. <CIN3	
		N	%	n	%	n	%	p	ptrend
**Age Category (Years)**	25–34	285	60%	22	52%	263	61%	0.47	0.74
	35–44	151	32%	18	43%	133	31%		
	45–54	37	8%	2	5%	35	8%		
	55+	3	1%	0	0%	3	1%		
**Number of Pregnancies**	0–2	199	42%	8	19%	191	44%	0.0002	0.0001
	3–4	179	38%	20	48%	159	37%		
	5–6	62	13%	5	12%	57	13%		
	≥7	31	7%	9	21%	22	5%		
**Number of Sexual Partners, Lifetime** †	1–2	145	30%	10	24%	135	31%	0.065	0.026
	3–4	145	30%	8	19%	137	32%		
	5–6	74	16%	10	24%	64	15%		
	≥7	105	22%	14	33%	91	21%		
**Marital Status**	Married	58	12%	7	17%	51	12%	0.42	n/a
	Unmarried, with partner	110	23%	12	29%	98	23%		
	Widowed	185	39%	16	38%	169	39%		
	Separated/Divorced	119	25%	7	17%	112	26%		
**Oral Contraceptive Use**	Never Used	408	88%	32	76%	376	87%	0.075	n/a
	Ever Used	57	12%	9	21%	48	11%		
**Number of Gynecologic Infections** *	0	123	26%	9	21%	114	26%	0.73	0.81
	1–2	322	68%	31	74%	291	67%		
	≥3	23	5%	1	2%	22	5%		
**Number of People in Residence**	1–2	149	31%	7	17%	142	33%	0.083	0.013
	3–4	92	19%	8	19%	84	19%		
	5–6	143	30%	14	33%	129	30%		
	≥7	84	18%	12	29%	72	17%		
**CD4 Category (per mm^3^)**	≥350	104	22%	7	17%	97	22%	0.76	0.61
	200–349	178	37%	17	40%	161	37%		
	<200	192	40%	17	40%	175	40%		
**Income (Rwandan Francs)**	<10K	166	35%	9	21%	157	36%	0.055	0.017
	>10K-≤35K	233	49%	22	52%	211	49%		
	>35K	66	14%	10	24%	56	13%		
**Current Health Insurance**	Yes	209	45%	23	55%	186	43%	0.19	n/a
	No	260	55%	19	45%	241	56%		
**Number of Meals with Meat (weekly)**	0	240	50%	15	36%	225	52%	0.1	0.14
	1–2	152	32%	19	45%	133	31%		
	≥3	74	16%	7	17%	67	15%		
**Malarial Infection**	Never	86	18%	3	7%	83	19%	0.096	0.047
	Past	205	43%	19	45%	186	43%		
	Recent	176	37%	20	48%	156	36%		
**HPV DNA Status**	Non-Carcinogenic	160	34%	4	10%	156	36%	<0.0001	<0.0001
	Carcinogenic HPV (excluding HPV16)	251	53%	24	57%	227	52%		
	HPV16	65	14%	14	33%	51	12%		
	Multiple HPV types	243	51%	27	64%	216	50%	0.077	n/a

*self-reported;

† includes incidences of rape; n/a = not applicable.

The results of the three logistic regression models are shown in **Table 2**. Of the 476 HPV+ women, 65 (14%) had HPV16, 251 (53%) had carcinogenic HPV other than HVP16, and 160 (34%) had only non-carcinogenic HPV. Among all HIV+/HPV+ women, factors significantly associated with a histologic diagnosis of CIN3+ included 7 or more (vs. 0-2) pregnancies (OR  = 8.7; CI 2.3-32.4), malarial infection in the previous six months (vs. never) (OR 3.9; CI 1.0-15.2), and 7 or more lifetime sexual partners (vs. 1–2 partners) (OR 2.8; CI 1.1-7.2). Compared to having non HPV16 carcinogenic genotypes, having HPV16 infection was positively associated (OR 2.6; CI 1.1-6.1) and having a non-carcinogenic HPV infection was inversely associated (OR 0.26; CI 0.09-0.81) with CIN3+ (vs. a less severe diagnosis).

**Table 2:** Multivariate associations of clinical and demographic characteristics with cervical intraepithelial neoplasia grade 3 or cancer versus less severe than CIN3 among 454 human immunodeficiency virus-infected, human papillomavirus (HPV)-infected women with complete data.

		Any HPV N = 454		Carcinogenic HPV n = 303		Non-HPV16 carcinogenic HPV n = 241	
		OR	95%CI	OR	95%CI	OR	95%CI
**Number of Pregnancies**							
	0–2 (ref)	1.00		1.00		1.00	
	3–4	2.58	1.00–6.64	2.27	0.85–6.05	**4.27**	**1.20**–**15.22**
	5–6	1.53	0.39–6.03	1.09	0.23–5.05	0.78	0.07–8.25
	≥7	**8.65**	**2.31**–**32.43**	**6.82**	**1.67**–**27.97**	**7.55**	**1.26**–**45.18**
**Number of Sexual Partners**							
	1–2	1.00		1.00		1.00	
	3–4	0.59	0.20–1.74	0.50	0.16–1.57	0.59	0.16–2.14
	5–6	1.87	0.65–5.37	1.31	0.41–4.13	1.14	0.25–5.19
	≥7	**2.76**	**1.06**–**7.20**	2.50	0.91–6.89	1.43	0.42–4.87
**CD4 Count**							
	per 100 cells/mm^3^	0.84	0.63–1.12	0.81	0.60–1.11	**0.62**	**0.40**–**0.97**
**Number of People in Residence**							
	1–2 (ref)	1.00		1.00		1.00	
	3–4	1.67	0.52–5.27	1.67	0.49–5.67	1.50	0.31–7.25
	5–6	1.39	0.47–4.17	1.22	0.38–3.95	1.61	0.37–7.01
	≥7	1.59	0.46–5.48	2.04	0.56–7.39	2.73	0.56–13.38
**Income (Rwandan Franc)**							
	≤10K (ref)	1.00		1.00		1.00	
	>10K-≤35K	1.55	0.63–3.78	1.37	0.53–3.53	1.07	0.34–3.41
	>35K	2.16	0.70–6.65	1.36	0.40–4.60	1.52	0.33–7.06
**Malarial Infection**							
	Never (ref)	1.00		1.00			
	Past	2.95	0.75–11.63	2.42	0.60–9.82	2.94	0.56–15.61
	Recent	3.86	0.98–15.24	3.26	0.80–13.34	2.35	0.44–12.48
**HPV DNA Status**							
	Non-Carcinogenic	**0.26**	**0.09**–**0.81**				
	Carcinogenic HPV (excluding HPV16)	1.00		1.00			
	HPV16	**2.62**	**1.12**–**6.12**	**2.93**	**1.24**–**6.89**		

OR = odds ratio; CI = 95% confidence interval; n/a = not applicable.

Analyses restricted to (all) carcinogenic HPV genotypes showed significant associations of CIN3+ with ≥7 pregnancies (vs. 0-2) and HPV16 (vs. non HPV16 carcinogenic genotypes). In analyses restricted to women with non-HPV16 carcinogenic HPV, ≥7 pregnancies (vs. 0-2) were associated with CIN3+. In both restricted analyses the magnitude of the associations of CIN3+ with 7 or more lifetime sexual partners and recent malarial infection were similar to that found in all HPV+ women, but were not statistically significant, perhaps because of the smaller sample sizes.

Both the magnitude (OR) and strength (p value) of the association of CD4 count with CIN3+ was greatest in the women with non-HPV16 carcinogenic HPV (Table 2): per 100 CD4 cells/µl increase, OR 0.62, CI 0.40-0.97, p = 0.035; OR 0.81, CI 0.60-1.11, p = 0.19; and OR 0.84, CI 0.63-1.12, p = 0.22 in women positive for non-HPV16 carcinogenic HPV, any carcinogenic HPV, and all HPV genotypes, respectively.

As in multivariate analysis using CIN3+ as the endpoint, CIN2+ was independently and positively associated with number of pregnancies (OR 5.88, CI 1.95-17.77 for ≥7 compared to 0-2), number of sexual partners (OR 2.60, CI 1.18-5.73), and inversely associated with non-carcinogenic HPV (OR 0.25, CI 0.10-0.58). Unlike in women with CIN3+, CIN2+ was not significantly associated with harboring HPV16 (OR 1.45, CI 0.68-3.08) or with recent malarial infection (OR 1.46, CI 0.59-3.59) (data shown in Supplemental online tables S1 and S2).

## Discussion

In this cross-sectional analysis of cervical histology in HIV+/HPV+ Rwandan women we found that immunosuppression as measured by CD4 count was significantly associated with CIN3+ only in women who harbored carcinogenic HPV genotypes other than HPV16. We and others have reported that HPV16 prevalence and incidence are not associated with CD4 count [12], [13], suggesting that HPV16 may be more able than other HPV genotypes to avoid immune surveillance in immune competent women. Immunosuppression may differentially affect the risk of progression to CIN3 and cancer of non-HPV16 carcinogenic HPV genotypes versus HPV16, perhaps mediated directly through the likelihood of HPV viral persistence. However, we had inadequate numbers (65) of women testing positive for HPV16 to allow direct analysis of the association of CD4 count with CIN3+ in HIV+ women with HPV16 genotype.

Compared to testing positive for non-HPV16 carcinogenic HPV, CIN3+ was inversely associated with testing positive for only non-carcinogenic HPV and positively associated with testing positive for HPV16. Notably, the fraction of women with CIN3+ who tested positive for HPV16 was less than expected (33% in this study versus 50–60% in other studies) and the strength of association and magnitude of the odds ratio for the association of HPV16 with CIN3+ were smaller than anticipated. This is further albeit indirect evidence that the relative effects of HPV16 in the development of cervical cancer may be less enhanced in HIV-infected compared to uninfected women, consistent with our prior report [12]. The other AIDS-defining cancers, Kaposi's Sarcoma and large B-cell lymphoma, have demonstrated dramatically decreased incidence since the advent of effective antiretroviral therapy (ART) [21], [22], which reverses much of the immune compromise of HIV infection. However, the HPV-related cancers, invasive cervical cancer and anal carcinoma, have in most reports not decreased in incidence in the era of effective ART [21]–[23]. This may be in part due to the lack of impact of immune suppression with HPV16 natural history, which in most populations causes at least 50% of cervical cancers.

We found that recent malarial infections had a significant positive association with CIN3+ in HIV+/HPV+ women. To our knowledge, this is the first report of any evidence that malarial infections might contribute to the risk of cervical precancer and cancer. The mechanisms by which malarial infections could increase the risk of CIN3+ are unknown, but might include additional challenges to the already compromised immune system of HIV+ women. HIV, HPV and malarial infections are highly prevalent in much of Africa, and any increased risk of CIN3+ associated with malaria could result in a substantial attributable risk. However, given the limited sample size in this analysis, this observation needs to be confirmed in other populations.

Several factors previously identified as associated with cervical precancer and cancer in general populations of women were also identified here in HIV+/HPV+ Rwandan women. We found that multiple pregnancies (7 or more) were associated with increased risk of CIN3+, independent of number of reported sexual partners. High parity has been reported as a risk factor for cancer in populations of unknown HIV serostatus [9]; the mechanism by which parity increases the risk is unknown but may be related to endogenous hormones causing further eversion of the squamocolumnar junction and/or tissue damage and inflammation resulting from birthing. A higher number of sexual partners was also associated with CIN3+, perhaps reflecting greater likelihood of multiple HPV exposures and thus stochastically to greater opportunity for HPV persistence and progression to CIN3+.

Our study has several limitations. It is a cross-sectional analysis and temporal or causal relationships cannot be inferred. The lack of follow-up does not allow observation of progression with specific HPV types. An additional limitation of this study is that HPV+ women without clinical indication of abnormal cytology or visual lesions were not colposcoped and therefore not biopsied. Thus visually unapparent, HPV+ high-grade lesions could have been missed [24], resulting in verification bias. We expect that this may have muted the strength of association by misclassifying some cases of CIN3+ as controls. Potential misclassification of women as HPV+ may have resulted from the use of CVL rather than tissue to determine HPV infection, thus eliminating from the population women who were in fact HPV infected. It may also have led to over-representation of non-carcinogenic HPV genotypes that preferentially infect the vagina [25], [26]. Further, cross-sectional detection of HPV is only a proxy for lifetime level of exposure. The smaller magnitude of the association of CIN3+ with CD4 count may not have been sufficient to achieve statistical significance because of the small number of women with HPV16. We also could not distinguish prevalent from incident infection. This may lead to underestimating the association of HR-HPV with CIN3 and cancer since prevalent infections represent a mixture of incident and persisting infections, the latter of which are on the causal pathway to cervical precancer and cancer.

In conclusion, we found that CIN3+ was inversely associated with CD4 cell count in HIV+ women co-infected with non-HPV16 carcinogenic HPV. The implication of these data is that a greater proportion of cervical precancer and cancer may be attributable to carcinogenic HPV genotypes other than HPV16 in HIV+ women compared to HIV-negative women but larger studies are needed to evaluate this directly. We also found some evidence that malarial infections may increase the risk of CIN3+; this warrants confirmation in larger population-based studies.

## Supporting Information

Table S1 Demographic and clinical characteristics in human immunodeficiency virus-infected, human papillomavirus (HPV)-infected women with and without cervical intraepithelial neoplasia grade 2 or more severe (CIN2+)
(0.11 MB DOC)

Table S2 Multivariate associations of clinical and demographic characteristics with cervical intraepithelial neoplasia grade 2 or more severe versus less severe than CIN2 among 454 human immunodeficiency virus-infected, human papillomavirus (HPV)-infected women with complete data.
(0.07 MB DOC)
