# Supplementary material for: Risk Factors for Cervical Precancer and Cancer in HIV-Infected, HPV-Positive Rwandan Women
Source: PLoS One. 2010 Oct 20;5(10):e13525. doi: 10.1371/journal.pone.0013525 (PMC2958122; doi:10.1371/journal.pone.0013525)
Supplement: Table S1 — Demographic and clinical characteristics in human immunodeficiency virus-infected, human papillomavirus (HPV)-infected women with and without cervical intraepithelial neoplasia grade 2 or more severe (CIN2+) (0.11 MB DOC) [file pone.0013525.s001.doc]

**Table S1. Demographic and clinical characteristics in** **human immunodeficiency virus-infected, human papillomavirus (HPV)-infected women with and without cervical intraepithelial neoplasia grade 2 or more severe (CIN**2+)

|  |  | **All (N = 476)** | | **CIN2+ (N = 63)** | | **<CIN2 (N = 413)** | | **CIN2+ vs. <CIN2** | |
| --- | --- | --- | --- | --- | --- | --- | --- | --- | --- |
|  |  | **N** | **%** | **n** | **%** | **n** | **%** | **p** | **ptrend** |
| **Age Category (Years)** | 25-34 | 285 | 60% | 29 | 46% | 256 | 62% | 0.020 | 0.22 |
|  | 35-44 | 151 | 32% | 31 | 49% | 120 | 29% |  |  |
|  | 45-54 | 37 | 8% | 3 | 5% | 34 | 8% |  |  |
|  | 55+ | 3 | 1% | 0 | 0% | 3 | 1% |  |  |
|  |  |  |  |  |  |  |  |  |  |
| **Number of Pregnancies** | 0-2 | 199 | 42% | 14 | 22% | 185 | 45% | 0.0001 | 0.0001 |
|  | 3-4 | 179 | 38% | 30 | 48% | 149 | 37% |  |  |
|  | 5-6 | 62 | 13% | 8 | 13% | 54 | 13% |  |  |
|  | ≥7 | 31 | 7% | 11 | 17% | 20 | 5% |  |  |
|  |  |  |  |  |  |  |  |  |  |
| **Number of Sexual Partners, Lifetime^†^** | 1-2 | 145 | 30% | 15 | 24% | 130 | 32% | 0.050 | 0.011 |
|  | 3-4 | 145 | 30% | 14 | 22% | 131 | 32% |  |  |
|  | 5-6 | 74 | 16% | 13 | 21% | 61 | 15% |  |  |
|  | ≥7 | 105 | 22% | 21 | 33% | 84 | 20% |  |  |
|  |  |  |  |  |  |  |  |  |  |
| **Marital Status** | Married | 58 | 12% | 9 | 14% | 49 | 12% | 0.79 | n/a |
|  | Unmarried with partner | 110 | 23% | 16 | 25% | 94 | 23% |  |  |
|  | Widowed | 185 | 39% | 25 | 40% | 160 | 39% |  |  |
|  | Separated/Divorced | 119 | 25% | 13 | 21% | 106 | 26% |  |  |
|  |  |  |  |  |  |  |  |  |  |
| **Oral Contraceptive Use** | Never Used | 408 | 88% | 49 | 79% | 359 | 89% | 0.036 | n/a |
|  | Ever Used | 57 | 12% | 13 | 21% | 44 | 11% |  |  |
|  |  |  |  |  |  |  |  |  |  |
| **Number of Gynecologic Infections** | 0 | 123 | 26% | 13 | 21% | 110 | 27% | 0.55 | 0.55 |
|  | 1-2 | 322 | 68% | 47 | 76% | 275 | 68% |  |  |
|  | ≥3 | 23 | 5% | 2 | 3% | 21 | 5% |  |  |
|  |  |  |  |  |  |  |  |  |  |
| **Number of People in Residence** | 1-2 | 149 | 31% | 11 | 18% | 138 | 34% | 0.016 | 0.0016 |
|  | 3-4 | 92 | 19% | 10 | 16% | 82 | 20% |  |  |
|  | 5-6 | 143 | 30% | 24 | 39% | 119 | 29% |  |  |
|  | ≥7 | 84 | 18% | 17 | 27% | 67 | 17% |  |  |
|  |  |  |  |  |  |  |  |  |  |
| **CD4 Category (per mm^3^)** | ≥350 | 104 | 22% | 12 | 19% | 92 | 22% | 0.91 | 0.66 |
|  | 200-349 | 178 | 37% | 24 | 39% | 154 | 37% |  |  |
|  | <200 | 192 | 40% | 26 | 42% | 166 | 40% |  |  |
|  |  |  |  |  |  |  |  |  |  |
| **Income (Rwandan Francs)** | <10K | 166 | 35% | 17 | 27% | 149 | 37% | 0.23 | 0.091 |
|  | >10K-≤35K | 233 | 49% | 33 | 53% | 200 | 50% |  |  |
|  | >35K | 66 | 14% | 12 | 19% | 54 | 13% |  |  |
|  |  |  |  |  |  |  |  |  |  |
| **Current Health Insurance** | Yes | 209 | 45% | 28 | 44% | 181 | 45% | 1.00 | n/a |
|  | No | 260 | 55% | 35 | 56% | 225 | 55% |  |  |
|  |  |  |  |  |  |  |  |  |  |
| **Number of Meals with Meat** | 0 | 240 | 50% | 31 | 50% | 209 | 52% | 0.88 | 0.99 |
| **(weekly)** | 1-2 | 152 | 32% | 22 | 35% | 130 | 32% |  |  |
|  | ≥3 | 74 | 16% | 9 | 15% | 65 | 16% |  |  |
|  |  |  |  |  |  |  |  |  |  |
| **Malarial Infection** | Never | 86 | 18% | 9 | 14% | 77 | 19% | 0.63 | 0.36 |
|  | Past | 205 | 43% | 28 | 44% | 177 | 44% |  |  |
|  | Recent | 176 | 37% | 26 | 41% | 150 | 37% |  |  |
|  |  |  |  |  |  |  |  |  |  |
| **HPV DNA Status** | Non-Carcinogenic | 160 | 34% | 7 | 11% | 153 | 37% | <0.0001 | <0.0001 |
|  | Carcinogenic HPV (excluding HPV16) | 251 | 53% | 41 | 65% | 210 | 51% |  |  |
|  | HPV16 | 65 | 14% | 15 | 24% | 50 | 12% |  |  |

*self-reported; ^†^includes incidences of rape; n/a = not applicable
