# Supplementary material for: Risk Factors for Cervical Precancer and Cancer in HIV-Infected, HPV-Positive Rwandan Women
Source: PLoS One. 2010 Oct 20;5(10):e13525. doi: 10.1371/journal.pone.0013525 (PMC2958122; doi:10.1371/journal.pone.0013525)
Supplement: Table S2 — Multivariate associations of clinical and demographic characteristics with cervical intraepithelial neoplasia grade 2 or more severe versus less severe than CIN2 among 454 human immunodeficiency virus-infected, human papillomavirus (HPV)-infected women with complete data. (0.07 MB DOC) [file pone.0013525.s002.doc]

**Table S2**. Multivariate associations of clinical and demographic characteristics with cervical intraepithelial neoplasia grade 2 or more severe versus less severe than CIN2 among 454 human immunodeficiency virus-infected, human papillomavirus (HPV)-infected women with complete data.

|  |  | **Any HPV** | | **Carcinogenic HPV** | | **Non-16 carcinogenic HPV** | |
| --- | --- | --- | --- | --- | --- | --- | --- |
|  |  | **n=454** | | **n=303** | | **n=241** | |
|  | | **OR** | **95%CI** | **OR** | **95%CI** | **OR** | **95%CI** |
| **Number of Pregnancies** | |  |  |  |  |  |  |
|  | 0-2 (ref) | 1.00 |  | 1.00 |  | 1.00 |  |
|  | 3-4 | **2.33** | **1.01-4.89** | **2.32** | **1.05-5.11** | **3.34** | **1.33-8.41** |
|  | 5-6 | 1.47 | 0.51-4.24 | 1.43 | 0.45-4.54 | 1.70 | 0.43-6.66 |
|  | ≥7 | **5.88** | **1.95-17.77** | **4.62** | **1.36-15.73** | **4.61** | **1.09-19.54** |
| **Number of Sexual Partners** | |  |  |  |  |  |  |
|  | 1-2 | 1.00 |  | 1.00 |  | 1.00 |  |
|  | 3-4 | 0.79 | 0.35-1.80 | 0.83 | 0.34-2.02 | 1.19 | 0.44-3.20 |
|  | 5-6 | 1.74 | 0.72-4.23 | 1.58 | 0.60-4.17 | 1.60 | 0.49-5.24 |
|  | ≥7 | **2.60** | **1.18-5.73** | **2.63** | **1.11-6.21** | 2.30 | 0.85-6.25 |
| **CD4 Count** | |  |  |  |  |  |  |
|  | per 100 cells/mm^3^ | 0.87 | 0.70-1.08 | 0.84 | 0.66-1.07 | 0.77 | 0.58-1.04 |
| **Number of People in Residence** | |  |  |  |  |  |  |
|  | 1-2 (ref) | 1.00 |  | 1.00 |  | 1.00 |  |
|  | 3-4 | 1.53 | 0.58-4.05 | 1.50 | 0.54-4.20 | 1.26 | 0.38-4.26 |
|  | 5-6 | 1.93 | 0.81-4.63 | 1.54 | 0.61-3.93 | 1.77 | 0.61-5.16 |
|  | ≥7 | 2.17 | 0.80-5.93 | 2.29 | 0.80-6.56 | 2.05 | 0.61-6.95 |
| **Income (Rwandan Franc)** | |  |  |  |  |  |  |
|  | ≤10K (ref) | 1.00 |  | 1.00 |  | 1.00 |  |
|  | >10K-≤35K | 1.14 | 0.57-2.27 | 1.24 | 0.58-2.63 | 1.20 | 0.50-2.86 |
|  | >35K | 2.16 | 0.46-2.94 | 1.03 | 0.37-2.87 | 1.16 | 0.34-4.00 |
| **Malarial Infection** | |  |  |  |  |  |  |
|  | Never (ref) | 1.00 |  | 1.00 |  |  |  |
|  | Past | 1.38 | 0.57-3.36 | 1.71 | 0.64-4.57 | 1.83 | 0.62-5.45 |
|  | Recent | 1.46 | 0.59-3.59 | 1.64 | 0.60-4.48 | 1.29 | 0.42-3.90 |
| **HPV DNA Status** | |  |  |  |  |  |  |
|  | Non-Carcinogenic | **0.25** | **0.10-0.58** |  |  |  |  |
|  | Carcinogenic HPV (excluding HPV16) | 1.00 |  | 1.00 |  |  |  |
|  | HPV16 | 1.45 | 0.68-3.08 | 1.48 | 0.70-3.16 |  |  |

OR=odds ratio; CI=confidence interval; n/a=not applicable
